# Treatment of Scleroderma-Related Microstomia Using Hyaluronic Acid: An Interventional Study

**DOI:** 10.3390/life13112176

**Published:** 2023-11-07

**Authors:** Ofir Shir-az, Ariel Berl, Din Mann, Biader Samih Bilal, Yair Levy, Avshalom Shalom

**Affiliations:** 1 Department of Plastic Surgery, Meir Medical Center, Kfar Saba 4428164, Israel; ofir.shiraz@gmail.com (O.S.-a.); dinmannlit@gmail.com (D.M.); biader.bilal@gmail.com (B.S.B.); avshalom.shalom@clalit.org.il (A.S.); 2 The Tel Aviv Faculty of Medicine, Tel Aviv University, Tel Aviv 6997801, Israel; levy.yair@clalit.org.il; 3 Department of Internal Medicine E, Meir Medical Center, Kfar Saba 4428164, Israel

**Keywords:** systemic sclerosis, microstomia, hyaluronic acid

## Abstract

Systemic sclerosis (SSc) or scleroderma is a rare, systemic, autoimmune connective tissue disease. It causes increased collagen synthesis, leading to multi-organ sclerosis, including the skin and joints. Patients’ overall health and quality of life are harmed dramatically. Involvement of the face and, especially, the oral opening can limit patients’ ability to speak and eat, oral hygiene, and cosmetic appearance. Profhilo^®^ (NAHYCO^®^) is an over-the-counter product consisting of pure hyaluronic acid. It is used to improve skin quality by increasing collagen production and adipocyte vitality. This interventional study evaluated the results of perioral injections of hyaluronic acid in terms of improved skin quality, elasticity, and increased oral opening. Patients diagnosed with SSc received an injection of one syringe of Profhilo^®^ (2 mL of hyaluronic acid) at each of two clinic visits at one-month intervals. The oral opening was measured between the upper and lower central incisors before and after treatment. Quality of life was assessed using the modified Rodnan Skin Score and Health Assessment Questionnaire-Disability Index. A total of 14 patients received the first treatment, and 11 received the second treatment. The mean oral opening increased from 31.6 mm (range 17–50 mm) prior to therapy to 35.8 mm (range 21–56) 2 months following the second injection. Statistical analysis showed that there was a significant increase in the oral opening as observed one week (36.2 mm, *p* = 0.011), one month (36.2 mm, *p* = 0.007), and three months (31.6 mm, *p* = 0.023) after the second injection, at the 5-month follow-up. Treatment of SSc patients’ perioral area with Profhilo^®^ can result in significant improvements in oral opening and quality of life.

## 1. Introduction

Systemic sclerosis or scleroderma is a rare, chronic, autoimmune disease characterized by a wide range of heterogeneous manifestations and symptoms involving the skin, internal organs, and the vascular system. Scleroderma commonly manifests as a chronic inflammatory response, presenting as skin ulcers, Raynaud’s phenomenon [1], myositis and synovitis, and generalized fibrosis, most notably affecting the skin and lungs [2]. The excessive deposition of the extracellular matrix leads to microvascular abnormalities and secondary Raynaud’s phenomenon, which may be seen in up to 90% of patients with systemic sclerosis [3,4]. Various modalities have been described to evaluate the microvascular system in order to differentiate between primary and secondary Raynaud’s phenomenon, as the secondary type syndrome can be an early presenting symptom of systemic sclerosis [3,4]. Studies have also tried to find correlations between disease severity, antibody detection, and increasing severity of disease, as measured by microvascular changes [5]. Treatments for secondary Raynaud’s may reduce severity but rarely resolve the clinical problem. However, early detection and intervention can be effective [3].

Two main subsets of systemic sclerosis have been described based on the extent of skin involvement and fibrosis. Diffuse cutaneous systemic sclerosis is characterized by fibrosis of the skin in the trunk and proximal parts of the limbs, whereas limited cutaneous systemic sclerosis involves the skin of the acral body parts, the face, and the limbs distal to the knees and elbows [6,7]. Patients with the diffuse cutaneous type have a poorer prognosis involvement of the skin and internal organs, which progresses quickly [6]. Systemic sclerosis is a relatively rare disease, with an estimated prevalence ranging from 3 to 47/100,000. It primarily affects middle-aged women [8,9,10,11].

The classic histological skin manifestations among patients with systemic sclerosis include increased thickening of the dermis and the subcutis, along with loss of peri adnexal fat and hair follicles and an infiltration of plasma cells and lymphocytes [12]. Other studies have shown variable changes, including atrophy of the epidermis, thickening of the epidermis, altered differentiation, and increased epidermal pigmentation [13,14,15]. Histopathological assessment of the skin of patients with systemic sclerosis has also been suggested as a useful adjunct to describe the clinical response to treatment [16].

As the autoimmune response in the pathogenesis of systemic sclerosis is still poorly understood, the mainstay of treatment for diffuse cutaneous systemic sclerosis is immunosuppression [17], including methotrexate, cyclophosphamide, azathioprine, and mycophenolate. Immunosuppression, with the goal of preventing progression and irreversible damage [18], is believed to have a role in reducing early disease activity, especially for the treatment of active lung, skin, or musculoskeletal manifestations [17].

The classic JAK-STAT and interferon pathways have received great attention due to their promising potential in repurposing targeted therapies for arthritis to systemic sclerosis [19,20]. In fibrosis-associated developmental pathways, BMP, Hedgehog, and PU.1 are expected to offer new targets to inhibit fibrosis [21]. However, a large body of evidence has indicated that the adaptive immune system, with autoreactive T cells and autoantibodies produced by B cells, has a central role in the pathogenesis of systemic sclerosis. In addition, inflammatory cytokines produced by innate immune cells have been detected in affected tissues of patients in the early and late stages of systemic sclerosis [22]. Currently, new therapeutic pathways are being studied, as the metabolism of systemic sclerosis is under constant investigation. Antioxidants as promotors of adipogenesis, adenosine deaminase, and cannabinoid receptor 2 agonist are among the possible therapeutic targets for fibrosis and systemic sclerosis [21].

Based on their immunosuppressive activity, regenerative capacity, and immune-privileged status, mesenchymal stem cells are considered by some to be attractive tools for cellular therapy in inflammatory diseases and have been investigated in clinical studies [23].

Regenerative processes and the scarring pathways are not yet fully understood, but the extra-cellular matrix, in particular, and hyaluronic acid have important roles in both tissue regeneration and pathological scarring [24].

The skin thickening seen in systemic sclerosis can have deleterious functional and cosmetic effects on patients, leading to a decrease in quality of life to the point where they are affected both physically and emotionally [25]. Hadj Said et al. conducted a review of 45 studies regarding orofacial manifestations of scleroderma that included a total of 328 patients. They found a diminished oral opening in the majority (69.8%) of patients [26]. The perioral fibrosis seen in scleroderma patients produces a characteristic microstomia together with microcheilia, causing severe difficulties in various aspects of daily life, such as consumption of food and beverages and oral hygiene.

Hyaluronan is an important, space-filling molecule due to its hydrophilic properties. The molecule is extremely hydrated and makes the extracellular matrix an ideal environment in which cells can move and proliferate. This polymer interacts with specific proteins called hyaladerins, such as TSG6, and membrane receptors like CD44, RHAMM, HARE, and toll-like receptor (TLR) 4/2, which modulate development, morphogenesis, tumorigenesis, migration, apoptosis, cell survival, and inflammation [27].

The biological roles of hyaluronic acid are dictated by the molecular weight and differential interactions with several cell surface-binding proteins, such as hyaladherins, including CD44. CD44 is one of the most well-characterized receptors of hyaluronic acid and is essential for cutaneous wound repair, where it regulates keratinocyte adhesion, motility, proliferation, differentiation, and survival. These processes are most likely achieved through an association with the actin cytoskeleton and downstream adaptor molecules [27]. The hyaluronic acid molecule is distributed widely throughout connective, epithelial, and neural tissues.

Recent findings support the notion that hyaluronic acid injections might alleviate inflammation [28]. Hyaluronic acid injections can also be used to treat atrophic facial acne scars [29] and as a possible treatment for skin fibrosis [24]. Hyaluronic acid is known for its capability to bind to water, allowing it to fill in gaps and spaces that moisturize and, thus, soften the skin.

Profhilo^®^ is a hyaluronic acid product consisting of a combined 32 mg of high molecular weight hyaluronic acid (1100–1400 kDa) and 32 mg of low molecular weight hyaluronic acid (80–100 kDa). It is the first product to achieve injectable 64 mg of hyaluronic acid in a 2 mL syringe. The low molecular weight hyaluronic acid in this hybrid complex is slowly released from the hybrid network and thereby prevents upregulation of the first inflammatory cytokines, effectively lowering the first “inflammation” phase and the overall inflammatory response. This, in turn, creates a positive light shock to “non-active” cells. Profhilo^®^ hybrid complexes were also found to protect the high molecular weight hyaluronic acid molecules by reducing the degradation of large hyaluronic acid chains (molecular weight of more than 1000 kDa) by more than 8-fold [30].

This study included patients with systemic sclerosis and severe microstomia. It investigated whether injections of hyaluronic acid (Profhilo^®^) to the perioral area would improve their skin quality by reducing fibrosis and improving elasticity, thereby leading to an increase in the oral opening and improved quality of life.

## 2. Materials and Methods

This prospective, interventional study was conducted from June 2020 through May 2021. All patients were referred to the outpatient Plastic and Reconstructive Surgery Clinic at Meir Medical Center by a certified rheumatologist, treating patients with systemic sclerosis or through social network groups. Inclusion criteria were patients with a diagnosis of systemic sclerosis documented by a certified rheumatologist, who were over the age of 18 years, and who consented to participate in the study.

Patients who were previously treated with autologous fat grafting underwent treatment with other hyaluronic acid products in the preceding year, or with a known allergy to hyaluronic acid, products were excluded from the study.

The treatment protocol included two injections to the perioral region during two separate visits, one month apart. Follow-up visits were conducted a week after each injection and at one month and three months after the second treatment.

### 2.1. Evaluations

The oral opening was measured, and photographs were taken at each visit. Measurements of the oral opening (in mm) included the distance between the maxillary and mandibular central incisors.

Quality of life was evaluated using the modified Rodnan Skin Score questionnaire and the Health Assessment Questionnaire-Disability Index. The modified Rodnan Skin Score questionnaire was used to evaluate the severity of skin fibrosis [31]. The modified Rodnan Skin Score divides a patient’s body into 10 sections, which include 4 areas in the upper limbs, 3 areas in the lower limbs, the chest, the stomach, and the face. A therapist assesses the severity of the fibrosis through a physical examination of each section. Each individual cutaneous area is scored from 0 to 3 (normal to severe).

The Health Assessment Questionnaire-Disability Index [32] (Appendix A) was completed by the patients during the first clinic visit prior to treatment and at the last follow-up visit. The modified Rodnan Skin Score was also assessed at these visits.

### 2.2. Procedures

Prior to the treatment injections, topical anesthesia was administered by applying Emla© cream, consisting of lidocaine 15% and prilocaine 5% to the lips. Regional anesthesia, infra-orbital, and inferior alveolar nerve blocks were performed by injecting 2 mL of lidocaine 2% with epinephrine 1:100,000 to the conventional anatomical locations. All treatments were administered by the first author (O.S.-A.).

Study data were stored anonymously and included demographics, oral opening (measured in millimeters), the Health Assessment Questionnaire-Disability Index, and the modified Rodnan Skin Score.

### 2.3. Data Analysis

Statistical analysis was performed using a questionnaire-based assessment of patient demographics, evaluation of treatment success, and comparison of the size of the oral opening before and after treatment. Univariate analysis was conducted with the chi-square test for dichotomous variables, Kruskal–Wallis and Mann–Whitney tests for ordinal variables, and paired *t*-test for numeric variables. A *p*-value of <0.05 was considered significant.

## 3. Results

A total of 14 patients were included in the study and received the first treatment. Eleven patients received the second treatment, of which nine completed 2 months of follow-up and four completed 5 months of follow-up.

All participants were female, with a mean age of 54.4 years (range 36 to 68 years). A total of 71.4% of the patients (10/14) had diffuse cutaneous scleroderma, and 4 (28.6%) had limited type scleroderma.

A rheumatologist specializing in scleroderma (Y.L.) evaluated the severity of the patients’ disease. Severity scores were assigned on a 1–10 (mild to severe) scale for each of the areas of general disease, skin involvement, lung involvement, skin ulcers, and gastrointestinal involvement (Table 1). Overall disease severity scores ranged from 9 to 2, with a mean of 6.5. Skin involvement severity scores ranged from a high of 9 to a low of 3. The mean value was 4.7.

The patients received a variety of therapies related to scleroderma. Five patients were treated with a single agent, including nintedanib, tocilizumab, methotrexate, or colchicine, and two patients received iloprost alone. The remaining patients were treated with a combination of mycophenolate mofetil and iloprost and/or bosentan or mintedanib, except for one who received methotrexate and iloprost (Table 1).

The mean total body modified Rodnan Skin Score before and after the treatments ranged from 0.4 to 1.66, with no statistically significant differences. The mean facial modified Rodnan Skin Score was 1.35 (range 0–2) at the first clinic visit before treatment and 0.75 (range 0–2) after the completion of two treatments (range 0–2). Evaluation of the specific facial modified Rodnan Skin Score before and after the follow-up period revealed no statistically significant difference (*p* = 0.569). Sixty percent (3/5) of the patients reported a 1-point decrease in the modified Rodnan Skin Score at their last visit.

The mean oral opening prior to therapy was 31.6 mm (range 17–50 mm). The mean oral opening one week after the first treatment was 33.1 mm (range 17–54 mm), and it was 34.8 mm before the second injection (one month after the first injection). Two months after the second injection, the mean oral opening was 35.8 mm (range 21–56). As measured from the mean baseline value of 31.6 mm, significant increases in the oral opening were observed one week (33.1 mm, *p* = 0.011) after the first treatment, one month (35.8 mm, *p* = 0.007) and three months (31.6, *p* = 0.023) after the second treatment (Table 2).

Evaluation of the increases in the oral opening was found to have a near significant negative correlation to microstomia severity after the first treatment (R = −0.51, *p* = 0.074), as well as after the second (R = −0.615, *p* = 0.058,) treatments. The increase in the oral opening was more pronounced the smaller the initial opening. Furthermore, the increases in the oral opening one and two months after the first treatment were not significantly correlated with the severity of the disease, severity of overall skin involvement, lung involvement, gastrointestinal involvement, or severity of skin ulcers (*p* > 0.1). The specific type of disease antibody (see Table 1) did not have a significant effect on the oral opening at one and two months after the injections (*p* > 0.1).

## 4. Discussion

Scleroderma is a chronic, immune-mediated disease with broad systemic effects, including skin fibrosis. Although the disease may involve internal organs, the damage to the skin is progressive, leading to severe functional disability with debilitating consequences on almost all aspects of daily activities and the patient’s quality of life. The severe functional impairment is manifested by decreased elasticity and conductivity of the skin, especially around the wrists and fingers and the perioral area.

The objective of this interventional study was to assess the effect of hyaluronic acid injections to the perioral area, delivered as Profhilo^®^, and improvements in the patients’ oral opening and quality of life.

The results of the study indicate that a series of two injections of Profhilo^®^ increased the oral opening from a mean of 31.6 mm prior to therapy to 35.8 mm two months after the second injection. A statistically significant improvement was observed one week, one month, and three months after the second treatment to the perioral area. We also noted an inverse correlation between the increased size of the oral opening before and after treatment compared to the severity of microstomia.

Quality of life measurements showed an almost statistically significant decrease in the mean facial modified Rodnan Skin Score before (1.35, range 0–2) and after (0.75, range 0–2) completion of two treatments (range 0–2), indicating improvement.

A systemic review of the literature characterized the prevalence of pathognomonic orofacial conditions in patients with systemic sclerosis. The lips were affected most frequently, with a prevalence of 57.6% (95% CI: 40.8–72.9%). The oral mucosa was involved in 35.5% of cases (95% CI: 15.7–62.0%) [33].

Many attempts have been made to improve the opening of the oral cavity in patients with scleroderma using non-surgical methods. These various non-surgical modalities and treatments to manage the facial and subcutaneous manifestations of scleroderma have resulted in varying rates of success [34]. Pizzo et al. [35] achieved a mean increase of 10.2 mm in the oral opening by developing an exercise program that included mouth stretching and oral augmentation exercises. Using intense pulse light, Comstedt and colleagues reported increases of approximately 1 mm per treatment in three patients [36]. This modality for the treatment of microstomia was also described by Rosholm et al., and although patients reported an increase in perioral mobility, a statistically significant increase in the oral opening was not found [37].

Due to its effect on muscle tone, injections of botulinum toxin to the perioral and facial areas have been suggested as a treatment option to improve facial appearance. Yet, this therapy was not found to have an effect on facial tissue atrophy [38]. Cumsky et al. [39] recently published their experience using a combination of hyaluronic acid filler and botulinum toxin. They achieved significant improvements in patients’ ability to drink, eat and retain food by 1 month after treatment. Tewari et al. studied the effect of phototherapy on microstomia in patients with systemic sclerosis. The study protocol was intense and included 40 treatment sessions. Published results were satisfactory, yet, a major limitation of this modality was the substantial number of treatments needed and the increased risk for developing skin cancer [40]. Kumar et al. described a case report where they used intradermal injections of hyaluronidase with various steroid supplements. They reported that the patient’s oral cavity opening increased but at the cost of side effects such as gastritis, among others [41]. Hyaluronidase injections into the mandible showed improvement in oral aperture, but this was achieved at the risk of severe discomfort at injection sites, along with angioedema and urticarial eruptions [42].

Autologous fat grafting for the treatment of facial manifestations among patients with scleroderma has also been described. Studies have shown an improvement in the aesthetic appearance, along with a significant effect on tissue elasticity and pliability. This modality has also been shown to increase the size of the oral opening [43]. The use of fat grafting over multiple procedures permitted the injection of increased volumes due to improved facial elasticity [43]. Evidence regarding fat grafting and improvements in the oral aperture is thought to be due to various mechanisms and tissue regeneration [44,45]. It is believed that the grafted adipose stem cells lead to an increase in the release of angiogenic factors with immunomodulatory effects [46,47]. Compared to non-permanent filler injections, fat grafts can provide structural support and encourage stem cell proliferation and differentiation [48].

Previous in vivo studies evaluated the effectiveness of treatment with Profhilo© to improve skin elasticity and the quality of wrinkles. Using photographic and 3D complexion analyses, significant improvements were reported among healthy patients, including a decrease in wrinkle depth and improved skin texture and hydration [49]. Furthermore, a statistically significant 25.1% increase in skin compliance and a 47.4% increase in skin elasticity were noted on cytometry analysis [49].

Hyaluronic acid has been shown to have profound effects on both the molecular and superficial aesthetic levels. Profhilo^®^ as hybrid (high and low molecular weight) hyaluronan cooperative complexes strongly affected the differentiation of adipose stem cells by up-regulating adipogenic genes and related proteins (leptin, PPAR-γ, LPL, and adiponectin). It was also found to have a positive effect on the proliferation of adipose stem cells [50].

It is important to identify the underlying problem that is the basis of the decreased oral aperture that occurs among patients with systemic sclerosis. As already known, scleroderma affects several types of tissues in various ways and is expressed differentially among patients. Several studies have used imaging modalities to assess microvascular involvement and their correlation to disease progression [3,4,5]. These studies focused on microvascular involvement, as seen in Raynaud’s phenomenon, which is a common symptom among patients with scleroderma. Imaging is sometimes the preferred method to evaluate tissue involvement. Information from histopathological examinations may contribute greatly to understanding the pathological processes affecting the various tissues and can help differentiate subgroups of patients according to the areas affected by the disease. However, in our experience, physical examination alone is sufficient when determining the appropriate treatment for patients with scleroderma. Future studies may benefit from the use of noninvasive imaging modalities to correlate disease progression and response to therapy.

We believe that injecting hyaluronan complexes into subdermal fat compartments on the anterior cheeks (especially medial to the mid-pupil line) and labial skin may recruit and differentiate stem cells in adipocytes. This resulted in a substantial improvement in the renewal of fat tissue, induced elastin production, and decreased fibrosis by altering collagen formations in the skin, as might happen when hyaluronic acid is injected into scars [51].

In the current study, we found that a series of two injections of Profhilo^®^ had a strong positive effect on increasing the oral aperture of patients with systemic sclerosis. A significant improvement was observed in the perioral area at one week, one month, and three months after the second treatment with Profhilo^®^.

The inverse correlation between the increase in the size of the oral opening before and after treatment compared to the severity of microstomia pretreatment reached near statistical significance. These results are in accordance with a previous study that demonstrated a relation between microstomia severity prior to autologous fat injections and improvement post-treatment [35]. The near-significant results could be the result of the small cohort included in this study. Further, large-scale studies are warranted to confirm and strengthen these findings.

A study that evaluated the severity of organ involvement among patients with diffuse type scleroderma found skin involvement in 233 of 953 (24%). Skin severity was evaluated using the modified Rodnan Skin Score [52]. Although skin involvement was studied in relation to the involvement of other organs, bone or joint involvement was not part of this study. A study by Scardina et al. [53] showed facial bone resorption in addition to facial skin hardening, and Benz et al. [33] found that the lips and oral mucosa had the highest prevalence of involvement among patients with systemic sclerosis. However, we were unable to find a study that included the bones and the temporomandibular joint in the sites affected by the disease. It is our hypothesis that a specific subgroup of patients might, in some yet unknown way, be affected by disease involvement of the bony perioral structures and joints. These patients present with a very decreased oral aperture, although both lips are very flexible with obvious laxity. Further investigation of these patients and the involvement of joints and bony structures is warranted.

Our results showed a significant increase in the oral opening one week (*p* = 0.011), one month (*p* = 0.007) and three months (*p* = 0.023) after the second Profhilo^®^ injection, along with decreases in the mean facial modified Rodnan Skin Score before (1.35, range 0–2), and after (0.75, range 0–2) completion of two treatments (range 0–2); yet, the change was not statistically significant. It should be noted that although the mean oral opening did not change from pretreatment compared to the 5-month follow-up (31.6 mm). This was due to the patients who dropped out. Focusing on the patients who completed the study, we noted that all had an increase in the oral aperture compared to their pretreatment values. We believe that the statistical trend would reach significance if the study were to be continued and the participation of a larger sample. This would further assess the effectiveness of the treatment for increasing the oral aperture and improving the modified Rodnan Skin Scores.

In the current study, one patient presented with severe microstomia (19 mm) along with a modified Rodnan Skin Score of 0 for lips and oral mucosa. A significant improvement was not achieved in the oral opening after one treatment, nor was there a subjective improvement in the softness of the perioral tissue. This led the patient to withdraw from the study. The same patient also expressed that she was severely disappointed due to previous encounters with dentists who were unable to provide a solution to her dental problems. Consequently, to prevent biases, we believe that this subset of patients who are severely affected (oral opening < 20 mm) should not be included in future studies.

In the study protocol presented here, the Profhilo^®^ injections were conducted under topical local anesthesia or a regional perioral block. In most cases, a perioral block was used during the second treatment for patients who wanted to avoid the pain that was experienced during the first treatment. It is our belief that the patients who experienced minimal improvement in the oral opening and who experienced pain during the first visit subsequently refused the second treatment, which may have led to their dropping out of the study.

This study had a few limitations as it was conducted in a single center and included a limited cohort. It also had a relatively short follow-up period and a fairly large drop-out rate. We noted an unexplained drop-out before the second treatment among three patients who had experienced a reasonably large improvement. This might be because they lived a far distance from the study center, experienced pain, or felt that the 374 improvement was sufficient and did not necessitate another treatment.

Further studies are warranted, and the methodology of perioral injections in this subset of patients should be evaluated. Among patients with scleroderma, tissue elasticity is inherently reduced. Therefore, the stretching incurred during perioral injections could lead to higher pain levels when compared to healthy patients receiving injections.

Decreasing the pain levels experienced at the first treatment might lead to higher satisfaction rates, encourage long-term follow-up, and increase adherence to the study protocol.

## 5. Conclusions

Treatment of scleroderma-induced microstomia using hyaluronic acid can produce significant and measurable improvements in the oral aperture and reported quality of life. It is important to identify patients who are suitable for treatment for decreased oral opening with high and low molecular weight hyaluronan complexes (Profhilo^®^). The results of this study introduce a novel modality that is reasonably safe and has minimal side effects. It adds to the armamentarium of physicians treating patients with systemic sclerosis who are affected by microstomia. Additional large-scale, multicenter research studies are required to corroborate these findings.

## Figures and Tables

**Table 1:** Baseline demographic characteristics.

Case	Age at Study (Years)	Age at Disease Onset	SSc Subtype (D/L)	Disease-Related	Antibody	SSc Severity (0–10)	Site/Organ Involvement Severity (0–10)				PHTN (Y/N)
							Skin Involvement Severity	Lung Involvement Severity	GI Involvement Severity	Ulcer Severity	
				Therapy							
1	68	---	D	Nintedanib	SCL70	8	3	7	2	0	Y
2	55	50	D	Tocilizumab	ARA POLY 3	9	3	6	9	0	Y
3	61	60	D	MMF, Iloprost	---	8	6	4	5	2	Y
4	36	30	D	---	SCL70	2	4	1	1	1	N
5	47	38	D	Methotrexate	SCL70	5	5	3	8	5	N
6	51	33	L	---	Centromere	5	4	0	3	5	N
7	60	45	L	Colchicine	Centromere	5	4	1	5	3	N
8	63	48	D	MMF, Iloprost, Bosentan	SCL70	6	4	5	5	0	N
9	50	44	D	MMF, Bosentan	SCL70	9	9	6	5	0	N
10	56	46	D	Iloprost	SCL70	6	6	5	2	0	N
11	46	37	D	MMF, Nintedanib	SCL70	9	9	5	7	6	N
12	68	51	L	Colchicine, Iloprost	Centromere	9	4	1	10	7	N
13	39	31	L	Iloprost	Centromere	6	3	2	4	9	N
14	62	53	D	Methotrexate, Iloprost	---	5	3	2	2	0	N
Mean ± SD	54.4 ± 9.6	43.5 ± 8.8	---	---	---	6.5	4.7	3.4	4.8	2.7	---

SSc—systemic sclerosis; D—diffuse cutaneous; L—limited cutaneous; GI—gastrointestinal; PHTN—pulmonary hypertension; Y—yes; N—no; MMF—mycophenolate mofetil.

**Table 2:** Measurements of oral openings throughout the study period *.

Case	Pretreatment			Oral Opening 1 Week after 1st Treatment	Oral Opening at 2nd Treatment	Oral Opening 1 Week after 2nd Treatment	2-Month Follow-Up		5-Month Follow-Up (from the First Treatment)			
	Oral Opening	MRSS (Face/Total Unilateral)					Oral Opening at 2 Months	Increase from 1st Measurement, mm (%)	Oral Opening at 5 Months	Increase from 1st Measurement, mm (%)	MRSS (Face/Total Unilateral)	
1	45	1	4	44	46	46	---	---	---	---	---	---
2	22	4	16	22	22	22	22	0 (0)	---	---	---	---
3	25	1	11	33	---	---	---	---	---	---	---	---
4	26	1	6	25	29	29	30	4 (15.4)	28	2 (7.7)	1	6
5	19	2	12	25	25	26	26	7 (36.8)	24	5 (26.3)	2	12
6	46	1	1	45	46	44	48	2 (4.4)	----	---	---	---
7	50	1	2	52	54	55	56	6 (12.0)	---	---	1	4
8	50	1	3	52	53	50	52	2 (4.0)	54	4 (8.0)	1	6
9	30	2	15	30	31.5	33	31	1 (3.3)	---	---	1	15
10	17	0	4	18	17	22	21	4 (23.5)	19	2 (11.8)	0	6
11	23	2	15	24	24	25	---	---	---	---	2	15
12	17	2	9	23	---	---	---	---	---	---	---	---
13	38	1	8	---	---	---	---	---	---	---	---	---
14	34	0	2	37	---	46	---	---	---	---	0	6
Mean	31.6	1	7.7	33.1	34.8	36.2	35.8	---	31.6	3.3 (13.4)	1	8.75

* Measurements are in mm. MRSS—Modified Rodnan Skin Score.

**Figure A1:**
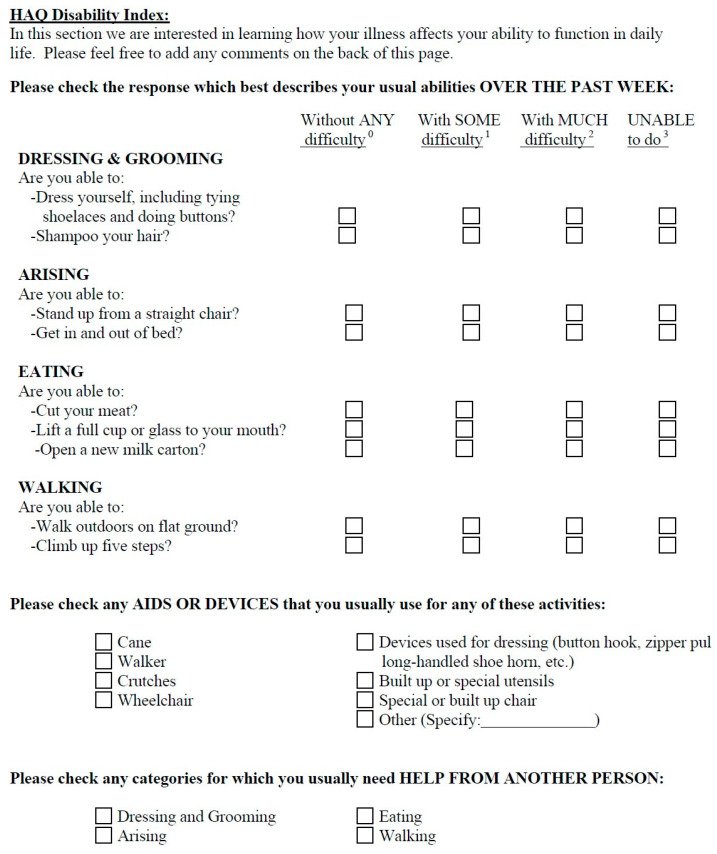

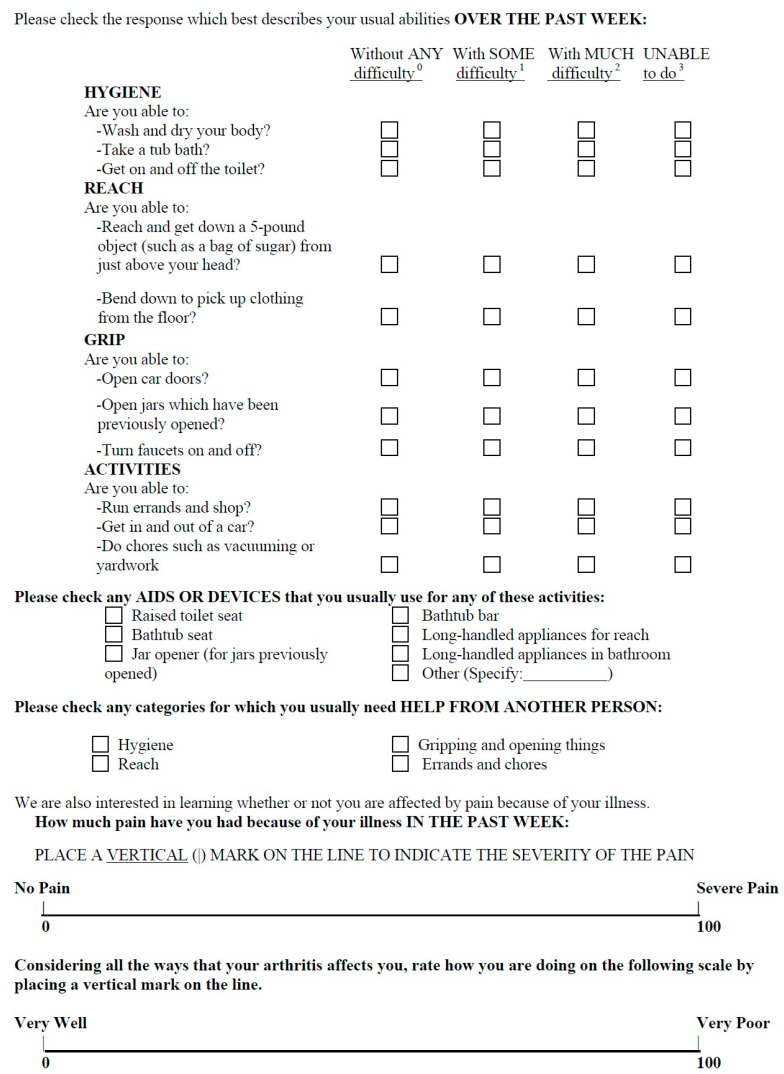
Health Assessment Questionnaire-Disability Index.

## Data Availability

The data presented in this study are available on request from the corresponding author. The data are not publicly available due to privacy reasons.
